# Ultra-Hypofractionated Stereotactic Body Radiotherapy for Localized Prostate Cancer: Clinical Outcomes, Patterns of Recurrence, Feasibility of Definitive Salvage Treatment, and Competing Oncological Risk

**DOI:** 10.3390/biomedicines10102446

**Published:** 2022-09-30

**Authors:** Marcin Miszczyk, Monika Szołtysik, Maja Hasterok, Gregor Goldner, Paweł Rajwa, Agnieszka Namysł-Kaletka, Aleksandra Napieralska, Małgorzata Kraszkiewicz, Małgorzata Stąpór-Fudzińska, Bartłomiej Tomasik, Grzegorz Woźniak, Grzegorz Głowacki, Konrad Kaminiów, Matthias Moll, Łukasz Magrowski, Wojciech Majewski

**Affiliations:** 1 IIIrd Radiotherapy and Chemotherapy Department, Maria Skłodowska-Curie National Research Institute of Oncology, Wybrzeże Armii Krajowej 15, 44-102 Gliwice, Poland; 2 Department of Radiation Oncology, Comprehensive Cancer Center, Medical University of Vienna, Währinger Gürtel 18-20, 1090 Vienna, Austria; 3 Department of Urology, Comprehensive Cancer Center, Medical University of Vienna, Währinger Gürtel 18-20, 1090 Vienna, Austria; 4 Department of Urology, Medical University of Silesia, 3-go Maja 13-15, 41-800 Zabrze, Poland; 5 Radiotherapy Department, Maria Skłodowska-Curie National Research Institute of Oncology, Wybrzeże Armii Krajowej 15, 44-102 Gliwice, Poland; 6 Treatment Planning Department, Maria Skłodowska-Curie National Research Institute of Oncology, Wybrzeże Armii Krajowej 15, 44-102 Gliwice, Poland; 7 Department of Oncology and Radiotherapy, Faculty of Medicine, Medical University of Gdańsk, Smoluchowskiego 17, 80-214 Gdansk, Poland

**Keywords:** prostate cancer, stereotactic body radiotherapy, radiotherapy, hypofractionated, survival, prognosis, second primary cancer, pattern of failure, salvage therapy

## Abstract

A cohort of 650 patients treated for localized prostate cancer (PCa) with CyberKnife^TM^ ultra-hypofractionated radiotherapy between 2011 and 2018 was retrospectively analyzed in terms of survival, patterns of failure, and outcomes of second-line definitive salvage therapies. The analysis was performed using survival analysis including the Kaplan–Meier method and Cox regression analysis. At a median follow-up of 49.4 months, the main pattern of failure was local–regional failure (7.4% in low-, and 13% in intermediate/high-risk group at five years), followed by distant metastases (3.6% in low-, and 6% in intermediate/high-risk group at five years). Five-year likelihood of developing a second malignancy was 7.3%; however, in the vast majority of the cases, the association with prior irradiation was unlikely. The 5-year overall survival was 90.2% in low-, and 88.8% in intermediate/high-risk patients. The independent prognostic factors for survival included age (HR 1.1; 95% CI 1.07–1.14) and occurrence of a second malignancy (HR 3.67; 95% CI 2.19–6.15). Definitive local salvage therapies were feasible in the majority of the patients with local–regional failure, and uncommonly in patients with distant metastases, with an estimated second-line progression free survival of 67.8% at two years. Competing oncological risks and age were significantly more important for patients’ survival compared to primary disease recurrence.

## 1. Introduction

Prostate cancer (PCa) continues to be the second most common oncological diagnosis in men worldwide, and the fifth leading cause of cancer-related deaths. However, up to 50% of diagnosed patients do not require immediate interventional treatment. Randomized clinical trials have shown that active surveillance (AS) does not compromise survival [1], while it significantly improves quality of life (QoL) and functional outcomes [2,3] in patients with localized disease. Current clinical guidelines recommend AS as the preferred method of treatment for patients with low-risk (LR) cancer, and as a viable option for favourable intermediate-risk group (IR) PCa [4]. Despite these findings, the clinical utilization of AS remains low, and coordinated initiatives are vital in improving the standards of care [5].

The scepticism can be associated with the observation that despite no (disease-specific) survival benefit of surgery or radiotherapy (RT) over AS, interventional treatment may be associated with a lower rate of progression and metastatic disease [1,6]. The high average lifespan conveys a need for both an efficient and effective modality in a long perspective and might contribute to a minor difference in clinical progression, eventually translating into significant survival difference over a longer course of follow-up (FU) [7]. This hypothetical equilibrium is challenged by the emerging utilization of stereotactic body radiotherapy (SBRT) for oligometastatic disease [8], especially with the aid of PET-PSMA [9], and multiple local salvage modalities [10], which can allow for definitive treatment in the setting of limited cancer recurrence or dissemination.

Among the available interventional approaches toward PCa management, ultra-hypofractionated RT (UHRT) could be an alternative for AS in LR, a standard of care in IR, and possibly even a therapeutic option in selected patients with more advanced PCa [11]. The five-fraction delivery of 36.25–40 Gy combines surgery-like short overall treatment time, high precision, and exceptional cost-effectiveness while remaining generally non-invasive (except for biopsy and optional fiducial placement). Compared to conventionally fractionated RT, UHRT also takes advantage of the low α/β ratio of PCa, which signalizes a high sensitivity to increases in fraction doses [12,13,14,15]. Prospective randomized clinical trials have shown non-inferiority of UHRT [16,17], which is supported by the real-world data showing an excellent toxicity profile, and as few as 3% clinical failures at a median FU of 31.3 months [18]. Moreover, as metastases-directed therapy and local salvage methods for local–regional recurrences have been considered a standard of care at our institution for over a decade, we recorded a significant subset of clinical failures treated with ‘second-line definitive treatment’.

In this publication, we aimed to analyze the patterns of recurrence, feasibility, and efficacy of local salvage therapies, and assess the competing oncological survival risk in patients treated with CyberKnife^TM^ UHRT for localized PCa.

## 2. Materials and Methods

This retrospective analysis included 650 patients consecutively treated between 2011 and 2018 at a single tertiary institution for localized PCa with primary CyberKnife^TM^ UHRT and androgen deprivation therapy (ADT) in applicable cases. The patients were treated and monitored according to a board-approved institutional protocol. The treatment was considered to be a standard of care; however, due to its novelty at the time of introduction (2011), the patients were treated and monitored in a manner similar to a prospective trial, and the outcomes were periodically assessed.

Patients considered for the treatment had to fulfil the following inclusion criteria: histopathological diagnosis of previously untreated prostate cancer, International Society of Urological Pathology (ISUP) grade of 2 or lower, TNM T stage of T2c or lower, TNM N and M stage 0, maximum prostate-specific antigen (PSA) concentration < 20 ng/mL, <50 mm maximum two-dimensional prostate measurement, and feasibility of implanting fiducials.

One patient with ISUP grade 3 was removed from the initial database due to concern for low variability in multivariable analysis. The maximum pre-treatment PSA exceeded 20 ng/mL in 10 patients (1.5%), which resulted in 10 patients being allocated to the high-risk group (HR). However, these patients were retained in the analysis assuming a proportional hazard of increase in PSA concentration.

Three Gold Anchor fiducials were implanted in a triangle-like configuration in each patient before treatment planning with computed tomography (CT) for real-time tracking of image-guided radiotherapy (IGRT), which was used for initial positioning and target tracking during each fraction. The patients were positioned on a vacuum mattress, with a moderately full bladder. The target volume and organs at risk were defined on the treatment planning CT, with the aid of a fused treatment planning Magnetic Resonance Imaging (MRI).

The patients received 36.25 Gy in five equal fractions of 7.25 Gy, delivered every other day on a CyberKnife^TM^ linear accelerator. Assuming an alpha/beta ratio of 1.5 for prostate cancer, this corresponds to an equivalent dose in 2 Gy fractions (EQD2) of 90.6 Gy, or a biologically effective dose (BED) of 211.5 Gy. The Clinical Target Volume (CTV) included the whole prostate and the proximal 1 cm of the seminal vesicles and was expanded by three mm posteriorly and five mm margin in the remaining directions to form the Planning Target Volume (PTV). None of the patients received pelvic lymph node irradiation. The use of androgen deprivation therapy (ADT) was discouraged in LR patients, while six-month ADT was suggested in IR patients with >50% positive biopsy cores or at least two of the following risk factors: PSA > 10 ng/mL, ISUP grade 2 and/or TNM T2b or T2c.

The clinical follow-up was based on the patients’ medical records. The follow-up visits were scheduled at 1, 4, and 8 months, and every 6 months thereafter. As described below, (18)F-fluorocholine-PET, later superseded by PET-PSMA and/or MRI, were considered as routine diagnostic modalities in case of rising PSA level and/or reasonable presumption of clinical failure. The complete survival data were available for each patient based on the National Cancer Registry. Each of the analyzed endpoints was calculated from the first day of RT until:

- Prostate, seminal vesicles or regional pelvic lymph node recurrence for local–regional control (LRC);
- Occurrence of distant metastases for freedom from distant metastases (FFDM);
- Distant metastases or death for metastases-free survival (MFS);
- Death for overall survival.

Or from the diagnosis of clinical failure (LRC, FFDM) to the occurrence of clinical progression or death after metastases-directed local therapy for second-line progression-free survival (sPFS).

Death was defined as all-cause mortality (non-disease-specific). Local–regional and distant metastases were defined according to the eight edition of TNM by AJCC. Biochemical failure was defined according to the Phoenix criterion (>2 ng/mL over nadir).

The Kaplan–Meier method was used for the estimation of survival curves. Cox regression analysis was used for survival analysis. The missing data included maximum PSA in one patient, and was substituted with a mean value for the purpose of Cox regression model. In the case of colinear variables (BC and LRC, BC and FFDM), only the more significant was chosen for the multivariable analysis. The *p*-value of <0.05 was considered to be statistically significant.

## 3. Results

The median medical history-based FU was 49.4 months (IQR 28.8–71.2). Complete survival data up to the day of data collection were available for all patients. The clinical characteristics of the study group are described in Table 1.

### 3.1. Local–Regional Control

**Figure 1:**
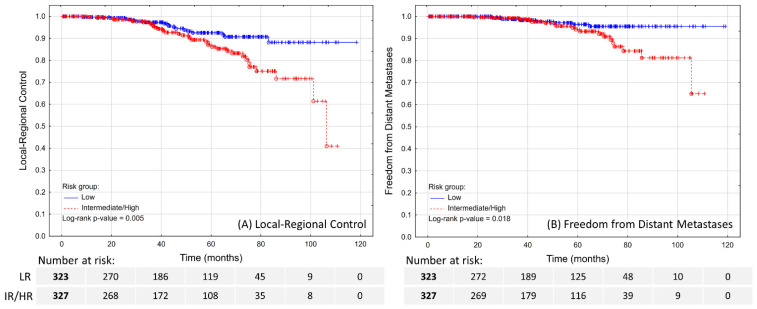
Local–regional control (**A**) and freedom from distant metastases (**B**) in patients treated with ultra-hypofractionated radiotherapy for localized prostate cancer.

The estimated 5-year LRC was 92.6% in LR patients and 87% in IR/HR patients (Figure 1A; *p* = 0.005). Over the course of FU, a total of 29 patients experienced a local recurrence, 21 had pelvic lymph node recurrence, and four had both. The diagnosis was made by PSMA-PET in the majority of the cases (32; 59.3%), followed by (18)F-fluorocholine-PET (16; 29.6%), MRI (5; 9.3%), and histopathological examination of material obtained through transurethral resection of the prostate in one case. In total, the local recurrence was confirmed histopathologically in 25 out of 33 applicable cases.

The local recurrences were diagnosed simultaneously or preceded by distant metastases in six cases. Out of the 23 isolated local recurrences, 13 patients were treated primarily with salvage brachytherapy (56.5%), 2 patients were treated with salvage radical prostatectomy (8.7%), and 1 patient was treated with salvage SBRT (4.3%). The remaining patients (7; 30.4%) received only ADT.

The regional lymph node recurrences were diagnosed along with distant metastases in six cases. The remaining 15 patients with isolated regional nodal recurrence were treated primarily with SBRT (13; 87%), or with ADT alone (2; 13.3%).

Finally, one of the four patients with simultaneous local and regional recurrence also had a distant failure. The remaining three patients received systemic treatment.

In total, 29 out of 41 patients (70.7%) with isolated local–regional failure received definitive treatment, which was combined with ADT in 11 cases.

### 3.2. Freedom from Distant Metastases

The estimated 5-year FFDM was 96.4% in LR patients and 94% in IR/HR patients (Figure 1B; *p* = 0.018). A total of 27 patients experienced distant metastases, which were localized in lymph nodes (7; 25.9%), bones (22; 81.5%), or lungs (2; 7.4%), including multifocal metastases in four cases. The distant metastases were diagnosed by PSMA-PET in majority of the cases (16; 59.3%), followed by (18)F-fluorocholine-PET (4; 14.8%), bone scan (4; 14.8%), CT (2; 7.4%), or MRI (1; 3.7%). These patients were treated with systemic therapy, and only four (14.8%) were found to be eligible and received SBRT as salvage treatment.

### 3.3. Second-Line Progression-Free Survival after Definitive Salvage Treatment

As described above, the first event regarded as a clinical failure was local recurrence in 23 cases, regional lymph node failure in 15 cases, simultaneous local–regional failure in three cases, and distant metastases in 18 cases. Out of these, 16/23 patients with local recurrence, 13/15 patients with regional lymph node recurrence, none with simultaneous local–regional recurrence, and 4/18 patients with distant metastases received definitive local treatment. During a median FU of 27 months (IQR 11.9–34.4), eight patients experienced clinical failure, which was manifested as local progression in the prostate in two cases, and distant metastases to either lymph nodes (*n* = 2) or bones (*n* = 4) in the remaining patients. The estimated 1- and 2-year sPFS were 86.3% and 67.8%, respectively.

### 3.4. Competing Oncological Survival Risk

There were 45 cases of malignant neoplasm (excluding non-melanoma skin cancers) diagnosed (*n* = 40) or recurred (*n* = 5) after the treatment for PCa, described in Table 2, resulting in an estimated 7.3% likelihood of a second malignant neoplasm diagnosis at 5-years after treatment. Considering the possible radiotherapy exposure, the time elapsed from PCa treatment to the diagnosis, and prior oncological medical history [19], 3/45 (6.7%) of these lesions could have been caused by the irradiation (Table S1), resulting in approximately 0.7% likelihood of possibly radiation-induced cancer at 5-years.

### 3.5. Metastases-Free Survival and Overall Survival

**Figure 2:**
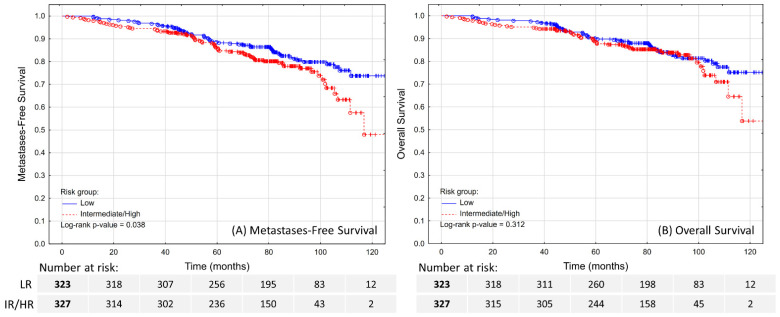
Metastases-free survival (**A**) and overall survival (**B**) in patients treated with ultra-hypofractionated radiotherapy for localized prostate cancer.

The estimated 5-year MFS was 88.6% in LR patients and 86.2% in IR/HR patients (Figure 2A; *p* = 0.038). No tested clinical features except for age (HR 1.1; 95% CI 1.07–1.14) were independent predictors of MFS (Table 3).

The estimated 5-year OS was 90.2% in LR patients and 88.8% in IR/HR patients (Figure 2B; *p* = 0.312). In the multivariable model (Table 3), only age (HR 1.1; 95% CI 1.07–1.14; *p* < 0.001) and diagnosis of a second malignant neoplasm (HR 3.67; 95% CI 2.19–6.15; *p* < 0.001) were significant prognostic factors for OS. Despite some evidence for negative impact on survival, distant failure did not reach the assumed statistical level of significance (HR 2.82; 95% CI 0.95–8.41; *p* = 0.062).

### 3.6. Exploratory Analysis

To account for the confounding factor of low cancer-specific mortality in LR patients, the survival analysis was repeated for selected subsets of patients including IR (*n* = 317) and HR (*n* = 10) cases. The results were consistent with the previously described findings, as shown in Table S2.

## 4. Discussion

Despite the uncontested impact of PCa on overall mortality, it should be appreciated that overdiagnosis and overtreatment can also be detrimental to public health. In this article, we have shown that both biochemical recurrence and clinical failures in patients treated for localized PCa with UHRT have a limited impact on survival, and local salvage is feasible in the majority of the local–regional recurrences. Most importantly, we have shown that age and competing oncological risks bear a significantly more important impact on survival, even in patients with an existing diagnosis of localized PCa, which can be a crucial consideration when deciding between AS and interventional treatment, and that the subsequent malignancies are, in vast majority of cases, unlikely to be associated with irradiation. Third, our treatment approach is associated with excellent short-term outcomes and could be an alternative if AS is not selected as a therapeutic option.

Mortality in PCa patients can be overshadowed by competing risks [6,20]. Despite low absolute rates, RT has been associated with a moderately increased risk of developing a second malignancy [21], including colon, rectal, and bladder cancers [22]. This, however, was only true for external beam RT, while odds of developing a second cancer were not increased in patients treated with brachytherapy [22]. Authors suggest that both exposure and approximately four to five years of delay is necessary to consider a malignancy to be secondary to irradiation [19,21]. As shown in Table S1, these criteria rule out the majority of reported malignancies as possibly secondary to irradiation. Moreover, it must also be considered that the CyberKnife^TM^ UHRT is a highly conformal local treatment [23]. Compared to the historic radiotherapy methods, the increased risk of secondary cancers might be lower due to limited exposure to organs at risk. Last but not least, some authors suggested that the incidence of secondary malignancies is comparable if adjusted for patients’ age and history of smoking [24]. However, a recent large cohort analysis showed that the risk remains increased after adjusting for both [21].

AS should be considered as the treatment of choice for the majority of PCa patients with localized disease, as it does not compromise survival while significantly improving quality of life [1,3]. However, interventional methods of treatment are not exclusive but complementary, as approximately half of the patients will eventually receive active treatment [1,25], and some will decline AS to start off with. In general, there are three ultra-short radiotherapy modalities that can be considered in such setting, including low-dose-rate brachytherapy (LDR-BT), high-dose-rate brachytherapy (HDR-BT), and UHRT. LDR-BT provides excellent results through the permanent implantation of radioactive seeds to the prostate [26]. HDR-BT utilizes a similar concept through the temporary injection of radiation sources to the prostate at the expense of two or more injections necessary to carry out the treatment [27]. Both are invasive and require anaesthesia, and only UHRT can be truly non-invasive. That said, in the majority of the cases, the treatment will still be associated with a simple minimally-invasive procedure of implanting fiducials to the prostate. It is important to consider that limitations associated with anatomy and comorbidities usually do not apply to UHRT, and patients disqualified from BT can often still be treated with UHRT.

UHRT is generally well tolerated, and in a previous publication regarding side effects, we have observed that over 90% and 75% of patients report no gastro-intestinal and genito–urinary toxicity, respectively [18]. LDR-BT and HDR-BT have also been shown to present favourable toxicity profiles [28,29]. An indirect comparison is difficult due to the differences in reporting low-grade events which constitute the vast majority of localized PCa treatment toxicity. Tsang et al. suggested that HDR BT is associated with less adverse effects compared to UHRT [30], and Widmar et al. found that UHRT causes more adverse effects compared to conventional fractionation. That said, a high-volume prospective comparison of LDR-BT, HDR-BT, and UHRT toxicity is lacking. Given the available data, the authors believe that all three methods present an acceptable toxicity profile, excellent treatment outcomes, and can be considered as equally viable treatment methods for localized LR or IR PCa patients [16,17,26,27].

Biochemical factors and local–regional failure are known to have insufficient correlation with survival [31]. While the excellent feasibility of post-RT local–regional recurrence salvage is confirmed by real-world data, the patients should be informed about a considerable risk of grade 3+ toxicity associated with the treatment of intra-prostatic local recurrences, and lack of clear survival benefit [10]. It is worth noting that due to the strict inclusion criteria and limited length of ADT as a part of radical treatment, our patients presented almost exclusively hormone-sensitive PCa (HSPC) distant failures. With the aid of rapidly developing systemic treatment options for HSPC, a significant survival benefit was achieved [32,33], likely contributing to the low impact of distant failures on survival in such patients.

While the authors believe that very few of the observed cancers could be associated with irradiation, the RT can indirectly interfere with the treatment of subsequent malignancies. For example, pelvic RT can limit the utilization of preoperative irradiation in rectal cancer or a bladder-preserving approach in bladder cancer patients. This could be a consideration when choosing an optimal treatment strategy for patients at increased risk of developing such cancers, for example, due to family history or known risk factors. Additional prognostic data might be useful for an optimal personalized approach while choosing candidates for treatment, including genomic profiling [34], medical imaging data [35], and verification of histopathology data at high-reference centres [36].

The authors acknowledge the limitations of the study. Despite the fact that the analysis was carried out based on a prospectively maintained cohort database, this should be regarded as a retrospective study. The selection of patients for a definitive salvage treatment was significantly biased by the institutional practices and protocols, as described in the article, and in approximately half of the cases, AS would be currently considered a standard of care. A longer observation period and larger study group could provide a more insightful view of the importance of distant failures and second(ary) cancers. Nevertheless, we believe that our findings are an important addition to the existing body of knowledge, and might provide an additional argument for de-escalating treatment in localized PCa patients.

## 5. Conclusions

The main pattern of failure after ultra-hypofractionated radiotherapy for localized prostate cancer is a local–regional failure, manifested at a similar rate as prostate or regional lymph node recurrence, followed by approximately twice less frequent distant metastases.

Definitive salvage treatment is possible in the majority of such patients with local–regional recurrences, and infrequently in patients with distant metastases. The definitive salvage treatment, however, is significantly less effective compared to primary treatment.

The independent prognostic factors for survival in patients treated with ultra-hypofractionated radiotherapy for localized prostate cancer included age and subsequent oncological diagnosis. There was no statistically significant association between survival and standard prognostic factors, biochemical factors, or local–regional failure. Despite some evidence for negative impact on survival, even distant failure did not reach the assumed level of significance.

Based on our results, competing oncological risks are significantly more important for survival than primary disease in patients treated with ultra-hypofractionated radiotherapy for localized prostate cancer, but very few of the subsequent malignancies can be regarded as possibly secondary to irradiation.

## Figures and Tables

**Table 1:** Clinical characteristics of the patients treated with stereotactic body radiotherapy for localized prostate cancer.

#	*n* = 650
Age (years) *	68.7 (64.2–74.4)
Clinical follow-up (months) *	47.2 (28.8–71.2)
PSA max (ng/mL) *	9.01 (6.08–10.92)
Histopathology:	
ISUP grade 1	524 (80.6%)
ISUP grade 2	126 (19.4%)
TNM:	
T1c	482 (74%)
T2a	64 (10%)
T2b	72 (11%)
T2c	32 (5%)
NCCN risk group:	
Low risk	323 (49.7%)
Intermediate risk	317 (48.8%)
High risk	10 (1.5%)
ADT (receiving):	350 (53.8%)
GnRH analogue + antiandrogen	249 (38.3%)
GnRH analogue	76 (11.7%)
Antiandrogen	25 (3.8%)
ADT duration [months] ^	11.99 (IQR 6.3–22.4)

* data presented as ‘median (IQR)’; ^ in patients receiving ADT.

**Table 2:** Second malignant neoplasms developed in patients treated with ultra-hypofractionated radiotherapy for localized prostate cancer over the course of follow-up.

Localization/Type:	Total Number	Time from Radiotherapy to Diagnosis (Months)
Lung cancer	11	7.1; 9.5; 9.5; 20.1; 36.8; 43.5; 53.6; 62.8; 63.2; 64.9 *; 71.5
Colorectal cancer	10	9.4; 10; 24.5; 28.4; 28.5; 34.5; 36.1; 46.9; 52.4 ^; 70.4
Renal cancer	7	12.9; 17; 18.2; 62.2; 67.6; 72.6; 94.8
Bladder cancer	6	20; 25.9; 27.7; 36.8; 38.9; 53.2 ^
Skin melanoma	3	42.5 *; 54.8
Gastro-intestinal stromal tumor	1	1.8 *
Pancreatic cancer	1	5.2 *
Multiple myeloma	1	6.4
Nasopharyngeal cancer	1	17.7 *
Ureter cancer	1	23.7
Mesothelioma	1	27.2
Gastric cancer	1	29.8
Breast cancer	1	65.3
Small intestine cancer	1	75.5 ^

* Recurrence of second cancer previously treated with definitive intent; ^ possibly a secondary cancer.

**Table 3:** Cox regression model for metastases-free and overall survival in patients treated with ultra-hypofractionated radiotherapy for localized prostate cancer.

Metastases-Free Survival				
	Univariable		Multivariable	
	HR (95% CI)	*p*-Value	HR (95% CI)	*p*-Value
Age	1.07 (1.04–1.1)	<0.001	1.07 (1.04–1.1)	<0.001
ISUP score	1.2 (0.76–1.91)	0.446	1.07 (0.67–1.71)	0.77
PSA max	1.03 (1–1.07)	0.091	1.03 (0.99–1.06)	0.12
TNM T	1.18 (0.98–1.41)	0.08	1.1 (0.92–1.32)	0.29
**Overall survival**				
	**Univariable**		**Multivariable**	
	**HR (95% CI)**	***p*-Value**	**HR (95% CI)**	***p*-Value**
Age	1.08 (1.04–1.11)	<0.001	1.1 (1.07–1.14)	<0.001
ISUP score	1.68 (1–2.84)	0.052	0.83 (0.48–1.43)	0.498
PSA max	1.04 (1–1.08)	0.086	1.02 (0.98–1.06)	0.293
TNM	1.09 (0.89–1.34)	0.379	1.01 (0.82–1.24)	0.939
Biochemical failure *	1.04 (0.54–2)	0.897		
Local–regional failure *	0.72 (0.34–1.55)	0.404	0.4 (0.12–1.3)	0.127
Distant failure *	1.76 (0.81–3.79)	0.151	2.82 (0.95–8.41)	0.062
Second subsequent malignant neoplasm	2.54 (1.55–4.17)	<0.001	3.67 (2.19–6.15)	<0.001

* Defined as respective BC, LRC and FFDM endpoints.; ISUP grade—International Society of Urological Pathology grade group; PSA max—maximum pre-treatment PSA concentration (ng/mL); TNM T—TNM T stage.

## Data Availability

Anonymized data available on request due to privacy and ethical restrictions.

## Supplementary Materials

The following supporting information can be downloaded at: www.mdpi.com/xxx/s1/.
