# Supplementary material for: Ultra-Hypofractionated Stereotactic Body Radiotherapy for Localized Prostate Cancer: Clinical Outcomes, Patterns of Recurrence, Feasibility of Definitive Salvage Treatment, and Competing Oncological Risk
Source: Biomedicines. 2022 Sep 30;10(10):2446. doi: 10.3390/biomedicines10102446 (PMC9598896; doi:10.3390/biomedicines10102446)
Supplement: Supplementary file 1 [file biomedicines-10-02446-s001.zip › biomedicines-1897997 sup materials proof.pdf]

Supplementary Materials – case-by-case analysis of subsequent malignant neoplasms after CyberKnife ultra-hypofractionated radiotherapy for localized prostate cancer.

Since the dose distribution of irradiation delivered during the treatment of prostate cancer was available for each patient, we have retrospectively assessed the dose delivered to the site where subsequent cancers developed. Then, we have rated each subsequent cancer diagnosis as ‚possible' or ‚unlikely' to be secondary cancer, based on the four principles (1-2):

1. Latency period (at least 4-5 years)
2. Radiation exposure
3. Different histological type to the original cancer
4. The second tumour was not evident at the time of radiotherapy

(1) Murray L, Henry A, Hoskin P, Siebert FA, Venselaar J; PROBATE group of GEC ESTRO. Second primary cancers after radiation for prostate cancer: a systematic review of the clinical data and impact of treatment technique. Radiother Oncol. 2014;110(2):213-228. doi:10.1016/j.radonc.2013.12.012

(2) Bagshaw HP, Arnow KD, Trickey AW, Leppert JT, Wren SM, Morris AM. Assessment of Second Primary Cancer Risk Among Men Receiving Primary Radiotherapy vs Surgery for the Treatment of Prostate Cancer. JAMA Netw Open. 2022;5(7):e2223025. doi:10.1001/jamanetworkopen.2022.23025

Table S1. Second malignant neoplasms developed in patients treated with ultra-hypofractionated radiotherapy for localized prostate cancer over the course of follow-up:

Localization / type:	Total number	Time from radiotherapy to diagnosis (months)
Lung cancer	11	7.1; 9.5; 9.5; 20.1; 36.8; 43.5; 53.6; 62.8; 63.2; 64.9*; 71.5
Colorectal cancer	10	9.4; 10; 24.5; 28.4; 28.5; 34.5; 36.1; 46.9; 52.4; 70.4
Renal cancer	7	12.9; 17; 18.2; 62.2; 67.6; 72.6; 94.8
Bladder cancer	6	20; 25.9; 27.7; 36.8; 38.9; 53.2
Skin melanoma	3	42.5*; 54.8
Gastro-intestinal stromal tumor	1	1.8*
Pancreatic cancer	1	5.2*
Multiple myeloma	1	6.4
Nasopharyngeal cancer	1	17.7*
Ureter cancer	1	23.7
Mesothelioma	1	27.2
Gastric cancer	1	29.8
Breast cancer	1	65.3
Small intestine cancer	1	75.5

*recurrence of second cancer previously treated with definitive intent.

Unlikely to be associated with irradiation:

1. Recurrent cancers, same histopathology as original cancer: lung cancer (64.9), skin melanoma (42.5), gastro-intestinal stromal tumor (1.8), pancreatic cancer (5.2) and nasopharyngeal cancer (17.7).

2. Low exposure (<1 Gy) and short interval: lung cancers (7.1; 9.5; 9.5; 20.1; 36.8; 43.5), renal cancer (12.9; 17; 18.2), colorectal cancers (28.5) mesothelioma (27.2), gastric cancer (29.8), ureter cancer (23.7), and multiple myeloma (6.4)

3. Low exposure (<1 Gy) but sufficient interval: lung cancers (53.6; 62.8; 63.2; 71.5), colorectal cancer (70.4), renal cancers (62.2; 67.6; 72.6; 94.8), melanoma (54.8) and breast cancer (65.3)

4. Considerable exposure but short interval: colorectal cancers (9.4; 10; 24.5; 28.4; 34.5; 36.1; 46.9) and bladder cancers (20; 25.9; 27.7; 36.8; 38.9)

Possibly associated with irradiation:

1. Small intestine cancer at 75.5 months (possible exposure, however cannot be assesed due to lack of imaging data – the cancer was treated at another hospital).

2. Bladder cancer at 53.2 months – high radiation exposure

3. Colorectal cancer at 52.4 months – located in the caecum, possible exposure of 4-5 Gy
