# Supplementary material for: Ultra-Hypofractionated Stereotactic Body Radiotherapy for Localized Prostate Cancer: Clinical Outcomes, Patterns of Recurrence, Feasibility of Definitive Salvage Treatment, and Competing Oncological Risk
Source: Biomedicines. 2022 Sep 30;10(10):2446. doi: 10.3390/biomedicines10102446 (PMC9598896; doi:10.3390/biomedicines10102446)
Supplement: Supplementary file 1 [file biomedicines-10-02446-s001.zip › biomedicines-1897997-Table S2.pdf]

Supplementary Materials:

Table S2. Cox regression model for overall survival in patients treated with ultra-hypofractionated radiotherapy for localized intermediate (n = 317) or high risk (n = 10) group prostate cancer.

# **(%)**	**Univariable**		**Multivariable**	
	**HR (95%CI)**	***p*-Value**	**HR (95% CI)**	***p*-Value**
Age	1.08 (1.03–1.12)	<0.001	1.09 (1.04–1.13)	<0.001
ISUP grade	0.88 (0.48–1.61)	0.683	0.83 (0.44–1.55)	0.555
PSA max	1.01 (0.97–1.06)	0.542	1.02 (0.98–1.08)	0.321
TNM T	0.99 (0.77–1.26)	0.916	0.94 (0.72–1.23)	0.672
Biochemical failure*	0.39 (0.12–1.25)	0.113		
Local-regional failure*	0.42 (0.13–1.36)	0.148	0.47 (0.11–1.92)	0.29
Distant failure*	0.89 (0.28–2.86)	0.844	1.56 (0.38–6.42)	0.54
Second subsequent malignant neoplasm	2.79 (1.39–5.57)	0.004	3.91 (1.9–8.07)	<0.001

*defined as respective BC, LRC and FFDM endpoints.; ISUP grade - International Society of Urological Pathology grade group; PSA max – maximum pre-treatment PSA concentration (ng/mL); TNM T – TNM T stage.
